# Rabbit Antithymocyte Globulin Induction in Heart Transplant Recipients at High Risk for Rejection

**DOI:** 10.31486/toj.20.0024

**Published:** 2021

**Authors:** Brent Kitto, Steven Thai, Brooke Baetz, Hamang M. Patel, Stacy A. Mandras, Sapna Desai, Selim R. Krim

**Affiliations:** ^1^ Department of Pharmacy, Ochsner Clinic Foundation, New Orleans, LA; ^2^ Section of Cardiomyopathy and Heart Transplantation, John Ochsner Heart and Vascular Institute, Ochsner Clinic Foundation, New Orleans, LA; ^3^ The University of Queensland Faculty of Medicine, Ochsner Clinical School, New Orleans, LA

**Keywords:** *Graft rejection*, *heart transplantation*, *immunosuppression*, *lymphocyte depletion*, *rabbit antithymocyte globulin*

## Abstract

**Background:** Induction with lymphocyte-depleting antibodies may improve allograft outcomes in heart transplant recipients who are at high immunologic risk for rejection.

**Methods:** We conducted a single-center retrospective cohort study that compared outcomes between adult patients receiving rabbit antithymocyte globulin (rATG) induction vs no induction from 2011 through 2017. Key exclusion criteria were patients who did not receive tacrolimus and mycophenolate and patients who did not meet high immunologic risk criteria.

**Results:** A total of 50 patients were included in the analysis. At 1 year, the composite primary outcome of ≥2R rejection as defined by the International Society for Heart and Lung Transplantation, any treated rejection, development of cardiac allograft vasculopathy, or graft loss was not different between groups (*P*=0.474). Serious infections were also similar between groups (*P*=0.963). In accordance with institutional guidelines, prednisone exposure was decreased in the rATG induction group at 1 month (24.04 mg ± 13.74 vs 35.18 mg ± 16.95; *P*=0.014).

**Conclusion:** These results suggest that while rATG induction does not improve heart allograft outcomes, it may enable reducing early corticosteroid exposure in patients at high immunologic risk.

## INTRODUCTION

Heart transplantation is an established treatment for end-stage heart failure. One-year survival approaches 90% compared to <50% with medical therapy alone.^1-3^ The success of heart transplantation is attributable in part to immunosuppression advances, such as the discovery of calcineurin inhibitors, that have dramatically improved survival.^4^ However, allograft dysfunction related to rejection and cardiac allograft vasculopathy (CAV) remains a significant issue.^1^

Induction with potent lymphocyte-depleting antibodies (LDAs) such as rabbit antithymocyte globulin (rATG) may prevent allograft dysfunction, particularly in patients at high risk of rejection. Compared to no induction, LDA use has been associated with reduced rejection and CAV.^5-10^ A study involving more than 2,000 heart transplant patients found that treated rejection occurred less in patients who received LDAs than in patients who did not (relative risk 0.76, 95% CI 0.68-0.85; *P*<0.01).^7^ However, LDAs are not universally used because of conflicting reports of efficacy.^11-13^ Further limiting their use is the concern for increased adverse effects related to infection.^6,7,13^ Finally, older studies that reported benefits from LDA induction included antiquated immunosuppression regimens, raising the question of whether LDA induction is beneficial when combined with newer immunosuppressive agents.^5,7^ Thus, the net benefit of LDA induction in high immunologic risk patients receiving contemporary immunosuppression requires further investigation.

Our hypothesis was that differences in clinical outcomes exist between patients at high immunologic risk who receive rATG induction vs no induction. Therefore, the objective of this study was to investigate if rATG induction was associated with improved heart allograft outcomes in patients at high immunologic risk receiving tacrolimus and mycophenolate. Safety outcomes, including infection and infusion reactions, were also assessed.

## METHODS

**Figure 1.**
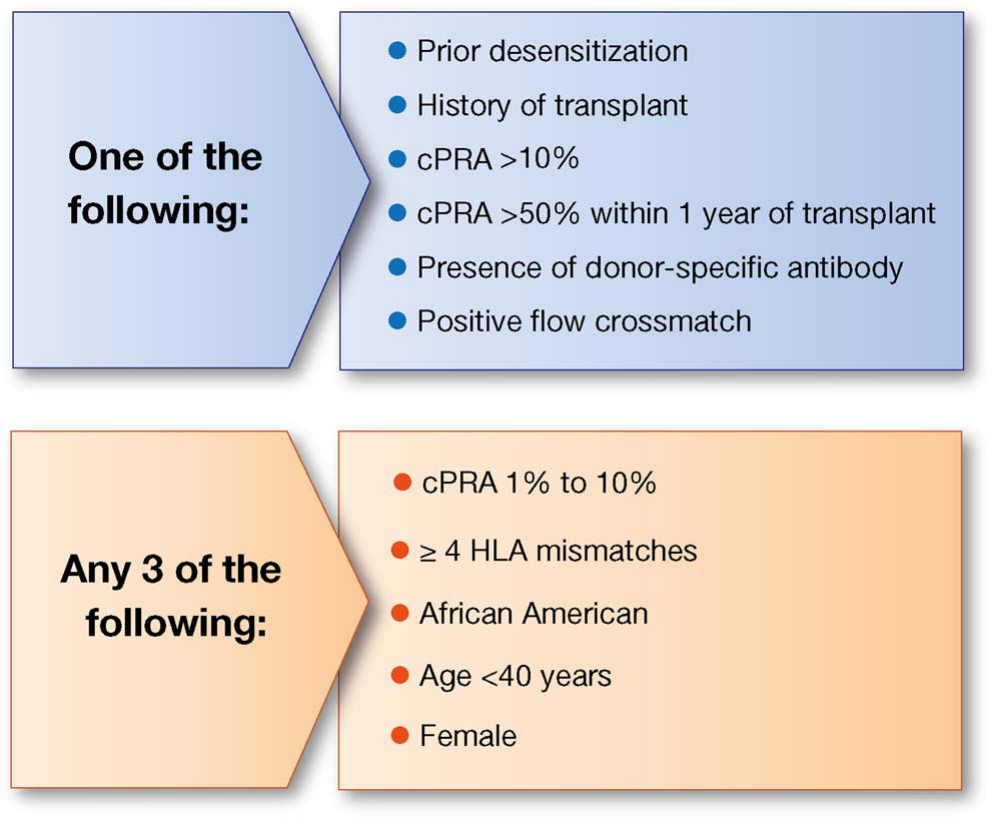
**High immunologic risk criteria.** cPRA, calculated panel reactive antibodies; HLA, human leukocyte antigen.

We conducted a single-center, retrospective cohort study comparing outcomes between patients receiving rATG induction vs no induction. The Ochsner Institutional Review Board approved the study protocol. Induction was defined as receiving rATG ≥3 mg/kg within 10 days of transplantation. Patients at least 18 years of age who received a heart transplant at our institution from September 2011 through November 2017 were eligible for inclusion. Patients were excluded if they received simultaneous dual organ transplantation, died within 48 hours, did not receive maintenance immunosuppression with tacrolimus and mycophenolate, or did not meet high immunologic risk criteria. High immunologic risk was defined as one of the following: prior desensitization, retransplantation, calculated panel reactive antibodies (cPRA) >10%, peak cPRA >50% within the year prior to transplant, pretransplant donor-specific antibody (DSA), or positive flow crossmatch, or as any 3 of the following: cPRA 1% to 10%, ≥4 human leukocyte antigen (HLA) mismatches, African American, age <40 years, or female sex (Figure 1).

The primary outcome assessed at 1 year was a composite of International Society for Heart and Lung Transplantation (ISHLT) ≥2R rejection, any treated rejection, development of CAV, and graft loss. Briefly, ISHLT defines acute rejection grades by the degree of lymphocyte infiltrate and myocardial injury present on endomyocardial biopsy. Mild, moderate, and severe rejection are designated 1R, 2R, and 3R, respectively.^14^ CAV was defined angiographically according to ISHLT criteria or by intravascular ultrasound demonstrating a ≥0.5 mm increase in maximal intimal thickness in any major coronary vessel at 1 year.^15,16^ Secondary outcomes were all-cause mortality, rATG infusion reactions (hypotension requiring discontinuation of infusion or serum sickness), posttransplant lymphoproliferative disease, and infections. Infections were defined by administration of intravenous antibacterial agents for ≥7 days or cytomegalovirus disease defined as DNAemia and fever ≥100.4 °F with at least one of the following: malaise, diarrhea, leukopenia <3,500/μL, or thrombocytopenia <100,000/μL.

Institutional guidelines prior to May 2014 recommended induction with rATG for patients with postoperative acute kidney injury. After May 2014, the guidelines were broadened to administer rATG to patients at high immunologic risk matching the previously described criteria. Intravenous rATG 0.75 to 1.5 mg/kg was administered once postoperatively and repeated daily to achieve 5 continuous days of lymphocyte suppression, defined as an absolute lymphocyte count <200 cells/μL or CD3 count <50 cells/μL. All patients received methylprednisolone 1,000 mg perioperatively, followed by a prednisone wean to either 20 mg/day at discharge if the patient received rATG or 40 mg/day if no rATG was given. Subsequently, prednisone was weaned per physician discretion with a maintenance dose goal of ≤5 mg/day by 6 to 12 months. Clinicians were encouraged to wean prednisone more rapidly in patients receiving rATG. Maintenance immunosuppression consisted of mycophenolate mofetil 3 g/day as tolerated and tacrolimus with a trough goal of 5 to 15 ng/mL. All patients were prescribed aspirin, statins, sulfamethoxazole-trimethoprim, and valganciclovir if at risk for cytomegalovirus disease. Serial endomyocardial biopsies were taken weekly during month 1, biweekly during month 2, monthly through month 6, and upon suspicion for rejection. Patients underwent routine coronary angiogram and intravascular ultrasound at 6 weeks and 12 months posttransplant.

Categorical variables were assessed using chi-square or Fisher exact test as appropriate. Continuous data were assessed using the *t* test or Wilcoxon-Mann-Whitney test. All statistical testing was 2-tailed. A *P* value <0.05 was considered statistically significant. SAS statistical software (SAS Institute Inc) for Microsoft Windows was used to conduct all analyses.

## RESULTS

**Figure 2.**
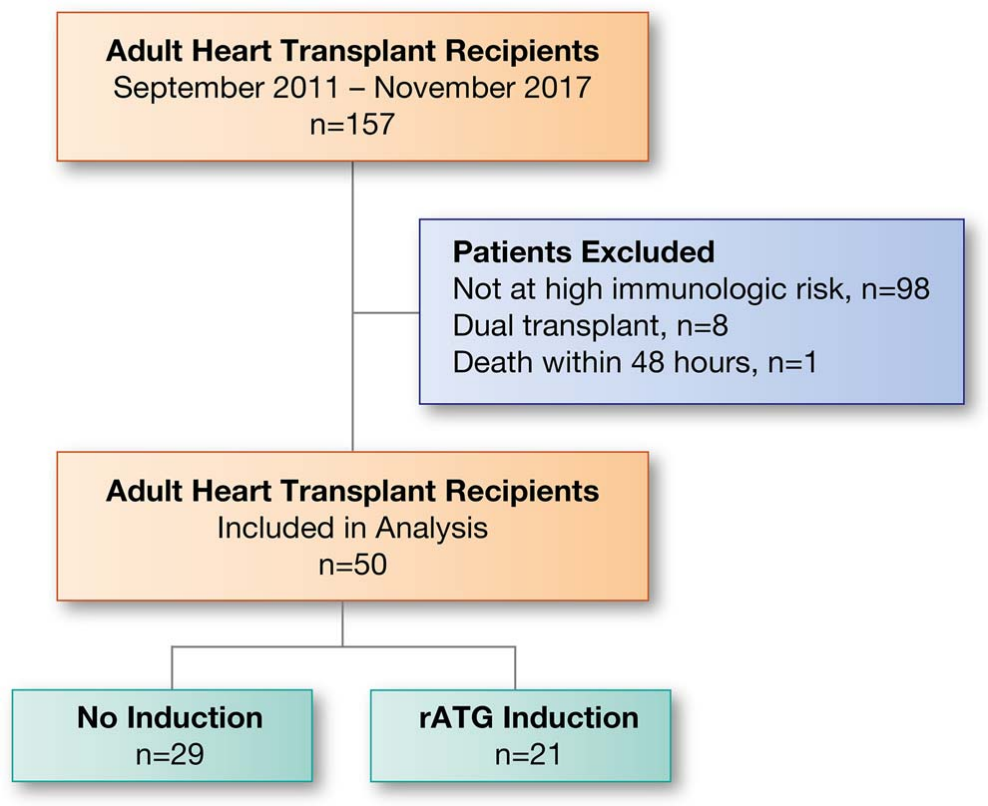
**Study participation.** rATG, rabbit antithymocyte globulin.

During the study period, 157 adult heart transplant recipients were screened. After exclusions, 21 patients were included in the rATG induction group, and 29 patients received no induction (Figure 2). Patient demographics and baseline clinical characteristics are described in Table 1. Most patients were African American, with a mean age of 46.7 years. Compared to the no-induction group, the rATG induction group had fewer females (58.6% vs 33.3%), patients with ischemic cardiomyopathy (27.6% vs 14.3%), and patients with diabetes prior to transplant (31.0% vs 14.3%), respectively, although none of these differences achieved statistical significance. By contrast, the percentage of patients at high risk for cytomegalovirus (donor seropositive/recipient seronegative) was significantly higher in the rATG induction group than in the no-induction group (38.1% vs 13.8%, respectively; *P*=0.047). Slightly more than half of the population in each group underwent left ventricular assist device implantation prior to transplant, and nearly all patients had ≥4 HLA mismatches. In general, the rATG induction group appeared to be more sensitized because of the higher percentage of patients with pretransplant DSA compared to the no-induction group (52.4% vs 37.9%; *P*=0.309), the significantly higher percentage of patients with a cPRA >25% (38.1% vs 10.3%; *P*=0.035), and the higher percentage of patients with a cPRA >50% in the year prior to transplant (28.6% vs 17.2%; *P*=0.491), respectively. Other than a lower prednisone dose at 1 month for patients in the rATG induction group vs patients in the no-induction group (24.04 mg ± 13.74 vs 35.18 mg ± 16.95, respectively; *P*=0.014), immunosuppression exposure was similar between groups (Table 2).

**Table 1.** Demographic and Clinical Characteristics

Variable	No-Induction Group (n=29)	rATG Induction Group (n=21)	*P* Value
Age, years, mean ± SD	46.7 ± 13.63	46.3 ± 17.77	0.941
Age <40 years	11 (37.9)	9 (42.9)	0.775
Female	17 (58.6)	7 (33.3)	0.093
African American	19 (65.5)	15 (71.4)	0.763
Ischemic cardiomyopathy	8 (27.6)	3 (14.3)	0.318
Pretransplant diabetes	9 (31.0)	3 (14.3)	0.201
Triglyceride level, mg/dL, mean ± SD	124.7 ± 73.32	99.5 ± 39.22	0.124
Prednisone			
6 months posttransplant	25 (86.2)	19 (90.5)	0.999
12 months posttransplant	20 (69.0)	16 (76.2)	0.708
Cytomegalovirus donor seropositive/recipient seronegative	4 (13.8)	8 (38.1)	0.047
Left ventricular assist device implantation	16 (55.2)	12 (57.1)	0.889
≥4 HLA mismatches	28 (96.6)	18 (85.7)	0.296
Pretransplant donor-specific antibody	11 (37.9)	11 (52.4)	0.309
cPRA 1%-10%	3 (10.3)	0	0.254
cPRA 11%-25%	3 (10.3)	2 (9.5)	0.999
cPRA >25%	3 (10.3)	8 (38.1)	0.035
cPRA >50% within previous year	5 (17.2)	6 (28.6)	0.491
History of transplant	0	0	
History of desensitization	0	1 (4.8)	0.420
Positive flow crossmatch	4 (13.8)	4 (19.0)	0.705

Note: Data are presented as n (%) unless otherwise indicated.

cPRA, calculated reactive panel antibody; HLA, human leukocyte antigen; rATG, rabbit antithymocyte globulin.

**Table 2.** Drug Levels and Doses

Drug	No-Induction Group (n=29)	rATG Induction Group (n=21)	*P* Value
Tacrolimus level, ng/mL, mean ± SD			
1 month	10.65 ± 3.090	10.14 ± 2.948	0.596
3 months	11.58 ± 4.741	11.23 ± 2.254	0.710
6 months	12.03 ± 3.505	10.81 ± 2.975	0.207
12 months	9.11 ± 3.227	8.19 ± 2.622	0.336
Mycophenolate daily dose, mg, mean ± SD			
1 month	2,740.7 ± 712.1	2,952.3 ± 218.2	0.389
3 months	2,192.3 ± 1,059	2,119.0 ± 960.5	0.938
6 months	1,907.5 ± 1,042.5	1,547.6 ± 1,011.2	0.183
12 months	1,773.5 ± 1,077.3	1,261.9 ± 956.8	0.056
Prednisone daily dose, mg, mean ± SD			
1 month	35.18 ± 16.95	24.04 ± 13.74	0.014
3 months	13.51 ± 8.35	12.09 ± 8.31	0.551
6 months	8.14 ± 7.89	4.78 ± 3.64	0.077
12 months	2.51 ± 2.09	3.28 ± 2.68	0.266

rATG, rabbit antithymocyte globulin.

Complete intravascular ultrasound and angiography data (at 6 weeks and 12 months) were available for 20 patients in the rATG induction group and 21 patients in the no-induction group. We found no difference in the composite primary outcome of ISHLT ≥2R rejection, any treated rejection, development of CAV, and graft loss (Table 3). The proportions of patients with any treated rejection (14.3% vs 31.0%) and CAV (25.0% vs 33.3%) were lower in the rATG induction group vs the no-induction group, although the differences were not statistically significant. Of the 12 total CAV cases, 4 were detected by both intravascular ultrasound and angiography, 7 by intravascular ultrasound only, and 1 by angiography only. Secondary outcomes were similar between groups. Nearly half of the study population had an infection within the year, but no infusion-related reactions or posttransplant lymphoproliferative disorder occurred. One death attributable to septic shock occurred in the no-induction group.

**Table 3.** Composite Primary Outcome and Secondary Outcomes

Outcome	No-Induction Group (n=29)	rATG Induction Group (n=21)	*P* Value
Composite primary outcome	14 (48.3)	8 (38.1)	0.474
>2R rejection^a^	3 (10.3)	2 (9.5)	0.999
Any treated rejection	9 (31.0)	3 (14.3)	0.171
Graft loss	0	0	
Cardiac allograft vasculopathy^b^	7 (33.3)	5 (25.0)	0.557
Secondary outcomes			
Posttransplant lymphoproliferative disorder	0	0	
Infusion reactions	0	0	
Infections	14 (48.3)	10 (47.6)	0.963
All-cause mortality	1 (3.4)	0	0.999

^a^Rejection as defined by the International Society for Heart and Lung Transplantation.

^b^Cardiac allograft vasculopathy is a composite outcome defined angiographically or by intravascular ultrasound. Complete data were available for 21 patients in the no-induction group and 20 patients in the rATG induction group.

Note: Data are presented as n (%).

rATG, rabbit antithymocyte globulin.

## DISCUSSION

Allograft dysfunction from rejection and CAV remains a significant barrier to heart transplant success. Patients with high immunologic risk features may be particularly susceptible to these negative outcomes.^17,18^ Potent immunosuppression with LDAs has been reported to reduce rejection and CAV. However, little data regarding this strategy exist in high immunologic risk populations receiving contemporary immunosuppression. Our results suggest that although safe, LDA induction with rATG did not reduce the composite endpoint of rejection, CAV, and graft loss in patients at high immunologic risk receiving tacrolimus and mycophenolate.

The net benefits of LDA induction vs no induction remain uncertain. Relevant studies have been retrospective in design, have used multiple formulations of LDAs, and have focused on rejection and mortality as endpoints. In brief, LDA induction is not consistently associated with reduced rejection and may increase the risk of infection.^7,11^ Moreover, reports from 2018 and 2019 suggest increased mortality with the use of LDA induction with rATG.^12,13^ Of interest will be the results of an ongoing pilot randomized study of LDA induction with rATG in heart transplantation (NCT03292861).^19^ Thus, as of May 2020, conflicting data supporting its use and its high cost explain why LDA induction is not universally adopted.

Nonetheless, patients at high immunologic risk are a subgroup that may derive benefit from enhanced immunosuppression with LDAs. Higgins et al conducted a database study that linked LDAs to improved survival among patients with a >5% chance of 1-year rejection-related mortality.^6^ By contrast, our study did not demonstrate a reduction in death or rejection with the use of rATG. Several possible reasons may account for the conflicting results. First, the Higgins et al study included a larger number of patients than our study, increasing the ability to detect a statistical difference. Second, although not reported, a significant number of patients in the Higgins et al study likely received cyclosporine and azathioprine given the study period of 1990-2001.^20^ Because of the greater immunosuppressive efficacy reported with tacrolimus and mycophenolate, which were used exclusively in our study, rATG induction may not have provided a meaningful incremental benefit in reducing poor outcomes related to rejection among our patients.^21,22^

Contrary to previous reports, rATG induction was not associated with reduced CAV in our study. Whereas most other studies defined CAV angiographically, our definition was a composite of 1-year intravascular ultrasound-detected increase in maximal intimal thickness ≥0.5 mm or angiographic CAV.^8,9^ The lack of benefit seen with rATG induction in our study may be attributed to the short follow-up time during which the rate of angiographic CAV detection is rare. Previous studies detected differences well beyond 1 year. In addition, detection bias may explain the seemingly neutral effects of rATG induction on CAV because more patients in the rATG induction group than in the no-induction group had complete intravascular ultrasound and angiogram data. In 2016, Azarbal et al conducted an intravascular ultrasound study that showed reduced coronary plaque thickening parameters with rATG. They found a trend toward less rapid intimal thickening, defined as a 1-year increase in maximal intimal thickness ≥0.5 mm.^10^ This definition is a robust surrogate for future adverse cardiac events and was present in 11/12 cases of CAV in our study. Although numerically lower in the rATG induction group, the difference was not significant.

A rationale for induction is reduced exposure to corticosteroids without increasing rejection. This strategy has been reported to be successful, but data are lacking for high immunologic risk populations.^23^ In our study, the rATG induction group had a reduced prednisone dose at 1 month with no increase in rejection. However, the difference in dose was no longer apparent after the first month, suggesting that rATG induction may enable safe corticosteroid reduction at least in the early posttransplant phase in high immunologic risk heart transplant recipients. Our study was not designed to assess adverse effects associated with corticosteroids. Thus, given the known metabolic adverse effects of corticosteroids, future studies should explore more aggressive corticosteroid reduction strategies, especially beyond the first month posttransplant in patients at high immunologic risk receiving LDA induction.

Although comparisons of LDA induction vs no induction exist, our study is salient because we included patients at high immunologic risk receiving tacrolimus and mycophenolate. We feel that our definition of high immunologic risk is valid, as it was based on risk factors derived from previous studies.^6,24^ Moreover, if major high immunologic risk criteria (ie, cPRA >10%, previous transplantation, desensitization, DSA, or positive crossmatch) were not met, 3 minor criteria (cPRA 1% to 10%, ≥4 HLA mismatches, African American, age <40 years, female) were required for the high immunologic risk designation and inclusion in the analysis. Nevertheless, our study has several important limitations. Aside from the known issues accompanying observational studies, the small number of patients in this study limited its statistical power. A larger sample size is likely required to detect a benefit of rATG induction, if a benefit truly exists. Second, antibody-mediated rejection and posttransplant antibodies were not included as endpoints although these outcomes would be of interest for patients at high immunologic risk. Third, the primary endpoint was a composite outcome of surrogate endpoints assessed at 1 year as representations of allograft outcome.

## CONCLUSION

In conclusion, rATG induction does not appear to improve heart allograft outcomes in patients at high immunologic risk receiving tacrolimus and mycophenolate. Consequentially, we cannot recommend the routine use of rATG induction in this unique population and suggest an individualized approach that optimizes the risk of immunologic allograft injury, the potential for adverse effects, and the increased drug costs incurred with induction. The use of rATG may permit the reduction of corticosteroid exposure. In a select group of high immunologic risk patients at risk for increased corticosteroid adverse effects, a reasonable strategy may be to administer rATG induction to reduce high doses of steroids used early after transplant. We hope our investigation stimulates the conduction of large, adequately powered studies that will determine whether rATG induction confers benefit to heart transplant patients at high immunologic risk.

## Notes

*This article meets the Accreditation Council for Graduate Medical Education and the American Board of Medical Specialties Maintenance of Certification competencies for Patient Care and Medical Knowledge.*
